# The Correlation Among COVID-19 Vaccine Acceptance, the Ability to Detect Fake News, and e-Health Literacy

**DOI:** 10.3928/24748307-20230621-01

**Published:** 2023-07

**Authors:** Abouzar Nazari, Maede Hoseinnia, Asiyeh Pirzadeh, Arash Salahshouri

## Abstract

**Background:**

The coronavirus disease 2019 (COVID-19) pandemic has seen a rise in the spread of misleading and deceptive information, leading to a negative impact on the acceptance of the COVID-19 vaccine and public opinion. To address this issue, the importance of public e-Health literacy cannot be overstated. It empowers individuals to effectively utilize information technology and combat the dissemination of inaccurate narratives.

**Objective:**

This study aimed to investigate the relationship between the ability to identify disingenuous news, electronic health literacy, and the inclination to receive the COVID-19 immunization.

**Methods:**

In this descriptive-analytical cross-sectional study conducted during summer 2021 in Isfahan, Iran, 522 individuals older than age 18 years, seeking medical attention at health centers, were surveyed. The participants were selected through a meticulous multistage cluster sampling process from the pool of individuals referred to these health centers. Along with demographic information, data collection instruments included the standard e-Health literacy questionnaire and a researcher-developed questionnaire designed to identify misinformation. The collected questionnaires were entered into SPSS 24 for statistical analysis, which included the Kruskal-Wallis test, the Chi-square test, the Spearman test, and logistic regression models.

**Key Results:**

The study findings revealed a statistically significant relationship between acceptance of the COVID-19 vaccine and the ability to identify deceptive news. An increase of one unit in the score for recognizing misinformation led to a 24% and 32% reduction in vaccine hesitancy and the intention to remain unvaccinated, respectively. Furthermore, a significant correlation was found between the intention to receive the vaccine and e-Health literacy, where an increase of one unit in e-Health literacy score corresponded to a 6% decrease in the intention to remain unvaccinated. Additionally, the study found a notable association between the ability to detect false and misleading information and e-Health literacy. Each additional point in e-Health literacy was associated with a 0.33% increase in the capacity to identify fake news (Spearman's R*ho* = 0.333, *p* < .001).

**Conclusion:**

The study outcomes demonstrate a positive correlation between the COVID-19 vaccine acceptance, the ability to identify counterfeit news, and proficiency in electronic health literacy. These findings provide a strong foundation for policymakers and health care practitioners to develop and implement strategies that counter the dissemination of spurious and deceitful information related to COVID-19 and COVID-19 immunization. [***HLRP: Health Literacy Research and Practice*. 2023;7(3):e130–e138.**]

## Plain Language Summary

We investigated the relationship between the ability to detect fake news and e-health literacy, with acceptance of a COVID-19 vaccine in Isfahan. The results demonstrated that acceptance of the COVID-19 vaccine is associated with a high level of ability to detect fake news and high level of e-Health literacy in individuals. Also, the high level of e-health literacy increases the ability to detection of fake news.

The coronavirus disease 2019 (COVID-19) is an acute respiratory syndrome that quickly led to a global pandemic and killed millions worldwide (Karlinsky & Kobak, 2021; Mathieu et al., 2020). Due to its high transmissibility and lethality, this disease has numerous health, social, and economic consequences affecting all countries (Moscadelli et al., 2020). COVID-19 can be described as “an epidemic once in a century” (Gates, 2020).

The COVID-19 epidemic was a serious threat to public health that could not be contained without the active participation of the public in adopting preventive behaviors, including vaccination (Chu et al., 2021). Vaccination is a powerful public health intervention to prevent infectious diseases (Thanh Le et al., 2020). Despite major advances in vaccination over the past century, this newly-emerged COVID-19 prompted the World Health Organization (WHO) to consider vaccine hesitancy a significant threat to global health (WHO, 2020). Even though the risk of the COVID-19 epidemic and its complications and death in the world and in Iran, some people have not yet accepted preventive behaviors such as vaccination (Yahaghi et al., 2021). This led to continued transmission of the mutated virus in communities, which resulted in mortality, making it more difficult to control transmission the virus (Larson et al., 2014). Therefore, there is a need to understand the factors to get vaccinated against COVID-19 (MacDonald, 2015).

Several studies have shown that false and misleading information are the main reasons for being doubtful about vaccination (Dubé et al., 2015; Lazarus et al., 2021; MacDonald, 2015; Puri et al., 2020). Fake news about COVID-19 began to spread at the beginning of the epidemic (van der Linden et al., 2020). Misinformation about health-related topics is a threat to public health (Ahmed et al., 2020). Fake news and misinformation on social media are often the causes of declining immunizations around the world (Chiou & Tucker, 2018; Dror et al., 2020; Puri et al., 2020), causing people to doubt the accuracy of the information about COVID-19 (Broadbent, 2019; Donzelli et al., 2018). At a time when people were exposed to a wealth of information on the internet and social media, it was essential to influence people to get the right information to control the epidemic (Bloomfield et al., 2021). Fake news is fabricated and deliberately intended to mislead or to deceive; fake news typically appears on sites that are masqueraded as genuine news outlets (Baptista & Gradim, 2022; Buckingham, 2019). The internet and social media are sources of alternative facts and fake news because there is not a sufficiently coherent fact-finding system on the internet. Real and fake news may be combined, and using the internet effectively depends on the user's ability to detect fake news and to filter it from a mass of other news sources (Tandoc et al., 2019). WHO and health authorities worldwide had been working closely with social media platforms to provide evidence-based information to the general public, trying to counter the misinformation circulating actively. This included cases of rumors about the COVID-19 vaccine spread through social media, such as the presence of microchips in the COVID-19 vaccine to monitor people, death after vaccination, or the high complications of vaccination. In Iran, there were some cases of fake news suggesting drinking alcoholic beverages to try to treat COVID-19.

Concerns over fake news had made it necessary to pay attention to various forms of media literacy, including e-health literacy (Jones-Jang et al., 2021). e-Health literacy is closely related to health behaviors especially in critical situations; therefore, e-Health literacy should be emphasized because although all individuals do not have a high level of health literacy during crisis, they can still search, acquire, and utilize health information through the internet, allowing them to adopt relevant health behaviors based on this information (Li et al., 2021; Norman & Skinner, 2006). However, if the impact of e-Health literacy is not considered, it may be insufficient to provide accurate and quality information to ensure optimal public health outcomes (Zarocostas, 2020). e-Health literacy is based on the concepts of both health and media literacy, which refers to an individual's ability to seek, understand, and appraise health information from electronic resources and make informed health decisions for addressing a health problem in everyday activities (Norman & Skinner, 2006); thus, allowing people seeking positive health information to better detect false and misleading information. Therefore, e-Health literacy is closely related to the acceptance and dissemination of fake news (Chong et al., 2020; Song et al., 2019). In general, many studies also have found that confusion about COVID-19 information is significantly higher among people with lower health literacy, leading to vaccine hesitancy (Hong et al., 2021; Okan et al., 2020; Spring, 2020). Previous studies separately examined fake news and e-Health literacy regarding COVID-19 (Brørs et al., 2020; Chong et al., 2020; X. Li & Liu, 2020). Research in France examined the relationship between the ability to detect fake news and health literacy with acceptance of a COVID-19 vaccine, and its results indicated that fake news and health literacy had a direct relationship with acceptance of the vaccine (Montagni et al., 2021). In Iran, several studies have highlighted the effect of misinformation on the epidemic control.

To date, the role of e-Health literacy and detecting fake news with acceptance of a COVID-19 vaccine has not been investigated in the literature in Iran. So, the present study aimed to determine the relationship between the ability to detect fake news and e-Health literacy with acceptance of a COVID-19 vaccine in Isfahan, Iran. The present study was conducted to test the hypotheses that the ability to detect false and misleading information and health literacy are each associated with whether individuals accept the COVID-19 vaccination.

## Methods

### Study Design and Sample

The present descriptive-analytical (cross-sectional) study examined 522 individuals older than age 18 years who visited health centers in Isfahan, Iran, from July 2021 to September 2021.

The sampling method had a two-stage cluster type. First, two health centers in Isfahan (Health Center 1 and Health Center 2) were selected for the case study. From that sampling, two comprehensive health service centers, Vali-e-Asr Comprehensive Health Service Center and Hazrat Ali Comprehensive Health Service Center, were randomly selected from the centers covered by Health Center 1; and two comprehensive health service centers, Fadaei Comprehensive Health Service Center and Amir Hamzeh Comprehensive Health Service Center, were randomly selected from the centers covered by Health Center 2. Using the convenience sampling method, all individuals who visited the centers based on inclusion criteria (people who were older than age 18 years, literate, and those who had not been vaccinated) were asked to complete the questionnaires, if desired.

### Data Gathering Process

Participants in this study completed a paper questionnaire. Data collection was stopped after 30 days due to the completion of the required number of participants. Participation was voluntarily.

Ethical considerations of the study included the confidentiality of information in the target population, obtaining a license from Isfahan University of Medical Sciences, a license from the provincial health center, and obtaining an informed consent from the participants. The study was approved by the Research Deputy of Isfahan University of Medical Sciences. In addition, The Ethical Committee of Isfahan University of Medical Sciences approved the study proposal (ID code: IR.MUI.RESEARCH.REC.1400.218; scientific code: 1400149). The required permission from the Education Department of Isfahan City was attained. Participation in the study was voluntary. Before taking part in the study, all participants provided written consent and the study goals were described.

### Study Instrument

The questionnaire required 10 to 15 minutes to complete, and it had three parts. The first section contained demographic questions (age, sex, education level, marital status, job, economic status, history of chronic illness, health work or education background, history of COVID-19, history of regular flu vaccination before COVID-19, history of hospitalization due to COVID-19, the rate of using social networks, the level of skill in using online networks, the source of news about COVID-19). The second part contained items related to a researcher-made questionnaire, which included the ability to detect fake news and was comprised of 12 questions. Some of the items on the questionnaire were: “The symptoms of COVID-19 are more severe than the common cold” or “All people infected with COVID-19 show the disease in appearance” with *true*, *false*, and *I do not know* options for a response. The *true* option received a score of 1, and the *false* and *I do not know* options received a score of zero. The ability to detect fake news was scored from 0 to 12. A higher score on the questionnaire meant the person was better (range, 0–12; with a median score of 3) able to detect false and misleading information.

A self-report questionnaire prepared by researchers (A.N., M.H., A.P.) was used to collect data. These questionnaires were developed based on a literature review to evaluate the ability to detect false and misleading information. The face and content validity of the questionnaire was approved by an expert panel including 10 health education professionals who were selected based on their competence and experience in the group with a CVR (Lawshe's content validity ratio) of 0.49 and CVI (content validity index) of 0.70. Its reliability was obtained using Cronbach's alpha coefficient (0.784).

The third questionnaire was the standard e-Health literacy questionnaire comprising eight items. The questionnaire was scored on a five-point Likert scale from *strongly agree* with a score 5 to *strongly disagree* with a score 1. The score range of the questionnaire was from 8 to 40. With higher scores (range, 1–40; with a median score of 28) implying higher e-Health literacy. The questionnaire was translated into Persian, and its validity and reliability of 0.88 (*p* < .00) were confirmed in a study by Bazm et al. (2016).

### Outcome Variable

Herein, the intention to get vaccinated against COVID-19 was the outcome variable. To measure the intention to get vaccinated, the following question was asked: “Are you going to get vaccinated against COVID-19?” There were three options of *no intention*, *yes*, and *doubtful*. It should be noted that the questionnaire was completed by the participants through the self-report method, and they were assured that their information would remain confidential.

### Data Analysis

The information obtained from the questionnaires were entered into SPSS 24 and were tested with the Chi-square test (for qualitative variables) and the Kruskal-Wallis test (for quantitative variables). To estimate the odds ratio in the *no intention* and *doubtful* groups compared with those who responded *yes*, we applied the multinomial logistic regression model at a significance level of 0.05.

## Results

A total of 522 individuals completed the questionnaires; of the 522, 160 (30.7%) did not intend to be vaccinated, 149 (28.5%) hesitated about the vaccination, and 213 (40.8%) intended to be vaccinated. The mean total score of detect fake news was 4.2 ± 99.42, and the mean score of e-Health literacy was 5.30 ± 26.70.

**Table 1** presents the demographic characteristics of the study population's e-Health literacy variables and the detection of fake news. **Table 2** shows the frequency of skills in using social networks, the source of information about COVID-19, detection of fake news, and e-Health literacy with the intention to get vaccinated against COVID-19.

**Table 1:**
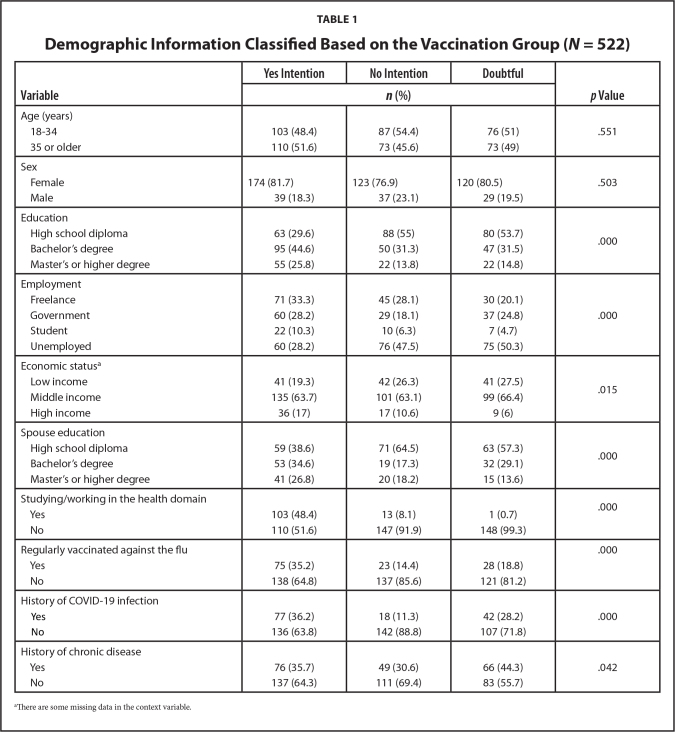
Demographic Information Classified Based on the Vaccination Group (*N* = 522)

**Variable**	**Yes Intention**	**No Intention**	**Doubtful**	***p* Value**

	***n* (%)**			

Age (years)				
18–34	103 (48.4)	87 (54.4)	76 (51)	.551
35 or older	110 (51.6)	73 (45.6)	73 (49)	

Sex				
Female	174 (81.7)	123 (76.9)	120 (80.5)	.503
Male	39 (18.3)	37 (23.1)	29 (19.5)	

Education				
High school diploma	63 (29.6)	88 (55)	80 (53.7)	.000
Bachelor's degree	95 (44.6)	50 (31.3)	47 (31.5)	
Master's or higher degree	55 (25.8)	22 (13.8)	22 (14.8)	

Employment				
Freelance	71 (33.3)	45 (28.1)	30 (20.1)	.000
Government	60 (28.2)	29 (18.1)	37 (24.8)	
Student	22 (10.3)	10 (6.3)	7 (4.7)	
Unemployed	60 (28.2)	76 (47.5)	75 (50.3)	

Economic status^a^				
Low income	41 (19.3)	42 (26.3)	41 (27.5)	.015
Middle income	135 (63.7)	101 (63.1)	99 (66.4)	
High income	36 (17)	17 (10.6)	9 (6)	

Spouse education				
High school diploma	59 (38.6)	71 (64.5)	63 (57.3)	.000
Bachelor's degree	53 (34.6)	19 (17.3)	32 (29.1)	
Master's or higher degree	41 (26.8)	20 (18.2)	15 (13.6)	

Studying/working in the health domain				
Yes	103 (48.4)	13 (8.1)	1 (0.7)	.000
No	110 (51.6)	147 (91.9)	148 (99.3)	

Regularly vaccinated against the flu				
Yes	75 (35.2)	23 (14.4)	28 (18.8)	.000
No	138 (64.8)	137 (85.6)	121 (81.2)	

History of COVID-19 infection				
Yes	77 (36.2)	18 (11.3)	42 (28.2)	.000
No	136 (63.8)	142 (88.8)	107 (71.8)	

History of chronic disease				
Yes	76 (35.7)	49 (30.6)	66 (44.3)	.042
No	137 (64.3)	111 (69.4)	83 (55.7)	

a There are some missing data in the context variable.

**Table 2:**
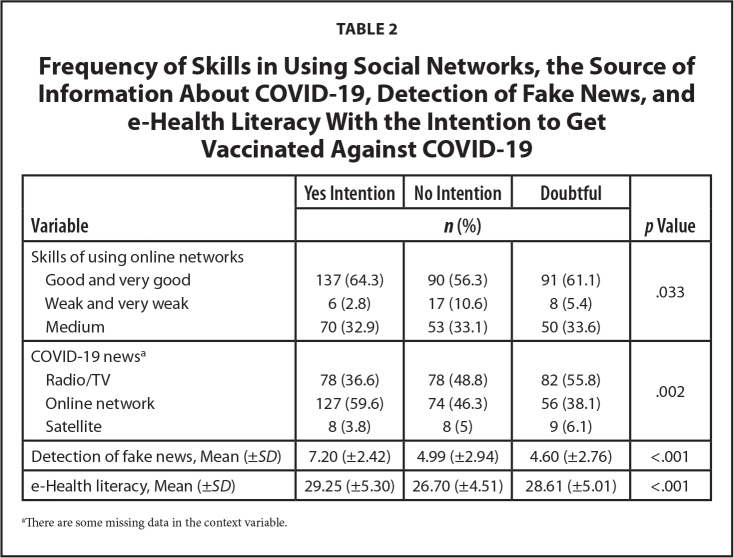
Frequency of Skills in Using Social Networks, the Source of Information About COVID-19, Detection of Fake News, and e-Health Literacy With the Intention to Get Vaccinated Against COVID-19

**Variable**	**Yes Intention**	**No Intention**	**Doubtful**	***p* Value**

	***n* (%)**			

Skills of using online networks				
Good and very good	137 (64.3)	90 (56.3)	91 (61.1)	.033
Weak and very weak	6 (2.8)	17 (10.6)	8 (5.4)	
Medium	70 (32.9)	53 (33.1)	50 (33.6)	

COVID-19 news^a^				
Radio/TV	78 (36.6)	78 (48.8)	82 (55.8)	.002
Online network	127 (59.6)	74 (46.3)	56 (38.1)	
Satellite	8 (3.8)	8 (5)	9 (6.1)	

Detection of fake news, Mean (±*SD*)	7.20 (±2.42)	4.99 (±2.94)	4.60 (±2.76)	<.001

e-Health literacy, Mean (±*SD*)	29.25 (±5.30)	26.70 (±4.51)	28.61 (±5.01)	<.001

a There are some missing data in the context variable.

The initial analysis revealed that there was a significant relationship between the intention to get vaccinated and the variables, namely education, employment, spouse's education, health work or education, regular flu vaccination, history of COVID-19 (*p* < .001), economic status (*p* = .015), chronic disease (*p* = .042), skills in using social networks (*p* = .033), and the source of information about COVID-19 (*p* = .002). The significance level was *p* < .05.

The results of the correlation between the ability to detect fake news and e-Health literacy demonstrated that there was a significant correlation between the two variables. Consequently, an increase in an e-Health literacy score enhanced the ability to detect fake news by 0.333% (Spearman's R*ho* = 0.333, *p* < .001)

**Table 3** represents the results of logistic regression. This model examined the relationship between the ability to detect fake news and e-Health literacy with intention to get vaccinated against COVID-19. Intention to vaccinate was considered as a basis. If the confidence interval contains the number one, it will be insignificant.

**Table 3:**
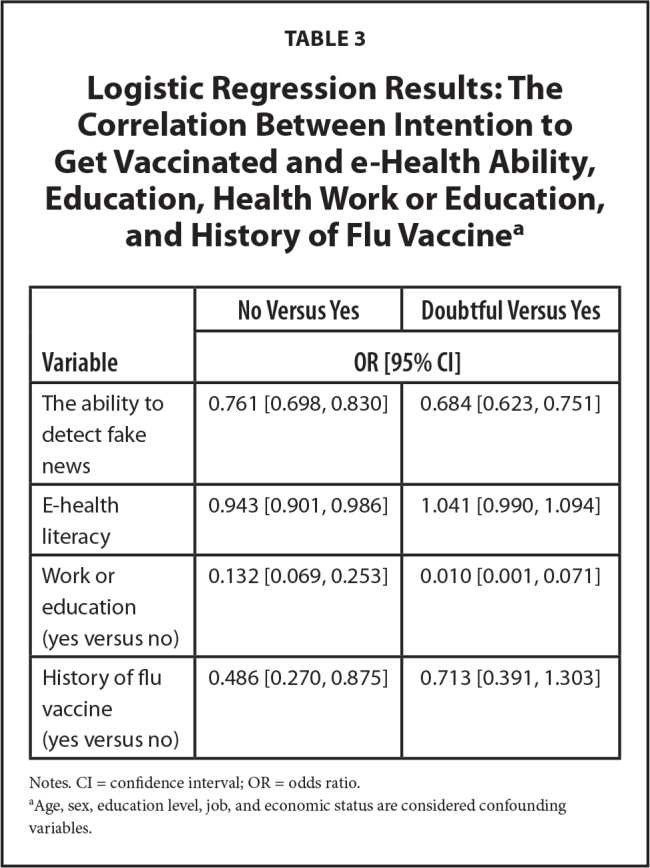
Logistic Regression Results: The Correlation Between Intention to Get Vaccinated and e-Health Ability, Education, Health Work or Education, and History of Flu Vaccine^a^

**Variable**	**No Versus Yes**	**Doubtful Versus Yes**
	**OR [95% CI]**	
The ability to detect fake news	0.761 [0.698, 0.830]	0.684 [0.623, 0.751]
E-health literacy	0.943 [0.901, 0.986]	1.041 [0.990, 1.094]
Work or education (yes versus no)	0.132 [0.069, 0.253]	0.010 [0.001, 0.071]
History of flu vaccine (yes versus no)	0.486 [0.270, 0.875]	0.713 [0.391, 1.303]

Notes. CI = confidence interval; OR = odds ratio.

a Age, sex, education level, job, and economic status are considered confounding variables.

**Table 3** illustrated that the intention to get vaccinated had a statistically significant correlation with the ability to detect fake news. Therefore, with a one-unit increase in the ability to detect fake news, *no intention* and *doubtful* in people decrease by about 24% and 32%, respectively. There was also a significant relationship between the intention to get vaccinated and e-Health literacy so that one-unit increase in e-Health literacy score reduced the *no* intention by 6%.

Also, there was a significant association between the intention to get vaccinated and health work or education. Participants working or studying in the health field were less likely to choose *no intention* (87%) and *doubtful* (99%) options than those who did not work in the health field. Participants working or studying in the health field were 78% less likely to select the *no intention* option and 99% less likely to select the *doubtful* option than those who did not work in the health field. So, most of them intended to get vaccinated. The *no intention* option was also lower in the participants who had a regular history of flu vaccination (51%).

The most challenging items in the questionnaire for the ability to detect fake news were “COVID-19 causes men to become infertile” with an average of 0.06 score and “a large number of people have died from the vaccine” with an average score of 0.10; most participants gave the wrong answers.

## Discussion

The present study aimed to determine the association between acceptance of a COVID-19 vaccine with the ability to detect fake news and e-Health literacy. For the first time in Iran, the present study evaluated the role of fake news and e-Health literacy together in accepting vaccination against COVID-19 aimed to examine whether the ability to detect fake news alters the intention to get vaccinated.

Based on the results, the ability to detect fake news and e-Health literacy both had a significant correlation with accepting the COVID-19 vaccine. The results of regression model analysis indicated that low e-Health literacy and low detection of fake news were both related to non-vaccination. The finding was consistent with a study in France. Their study revealed that people able to detect fake news better, unlike those with low e-Health literacy, were more likely to be vaccinated (Montagni et al., 2021). However, in the France study, public health literacy was examined and e-Health literacy was not examined.

The present research also demonstrated that ability to detect fake news is effective in the intention to get vaccinated. It is inferred that those with a lower ability to detect false and misleading information easily accept any news without scientific support and a reliable source, thereby reducing the intention to vaccinate. Numerous studies have shown that fake news spread much faster than credible sources, indicating the power of false and misleading information in influencing vaccination (Aquino et al., 2017; Dubé et al., 2015). For instance, Carrieri et al. (2019) studied vaccine hesitancy and the results indicated that the spread of this news led to a decrease in the immunization rate. A study in the United Kingdom (Freeman et al., 2022) also shed light on the correlation between fake news about COVID-19 and vaccine hesitancy and the results showed that misinformation about vaccination was associated with a reluctance to be vaccinated.

According to the findings, those with higher e-Health literacy were more likely to get vaccinated. This suggests that the ability to obtain information from a reliable source directly results in vaccination. Previous research has shown that health literacy is associated with vaccination intent and helps identify fake news. Previous studies have also stated that health literacy was associated with the intention to get vaccinated (Turhan et al., 2022; Wang et al., 2018) and helped to detect fake news (An et al., 2021; Jones-Jang et al., 2021). A study in Germany found that one-half of people older than age 16 years had insufficient health literacy and also observed a positive effect of health literacy on vaccination preferences (Okan et al., 2020). A study in Netherlands revealed that only parents with low levels of health literacy were willing to be vaccinated (Veldwijk et al., 2015) but in Germany and Netherlands studies, only health literacy was examined.

According to the findings in the present study, the average e-Health literacy was higher than the average ability to detect fake news. A possible explanation may be that the e-Health literacy questions were not difficult, and the individuals overestimated their abilities.

Results showed that there was a significant relationship between the ability to detect fake news and e-Health literacy. One-unit increase in e-Health literacy score enhanced the ability to detect fake news, and the two variables were directly correlated. However, a study in France did not find any significant relationship between the ability to detect fake news and health literacy (Montagni et al., 2021). This may be because the current study evaluated e-Health literacy, and the Montagni et al. (2021) study evaluated health literacy. There were also differences between the tools of measuring the ability to detect fake news and health literacy with the scales of the research.

A key point confirmed in other studies is the relationship between education and intention to get vaccinated (Hacquin et al., 2020; Lin et al., 2020). This study showed that higher education is associated with the intention to vaccinate. This may be because higher education contributes to greater awareness and higher health literacy. Individuals with higher education may have greater discernment in detecting fake COVID-19 news. Additionally, in the present study, the level of the spouse's education was effective in the intention to vaccinate. The reason could be that the spouse was an effective interpersonal support in influencing health behavior (Kreps et al., 2020). Education should be considered as an important factor in identifying news.

The results of the regression model analysis showed that people working or studying in the field of health were more likely to get vaccinated (Montagni et al., 2021). The impact of academic literacy on health and receiving up-to-date and trusted information from reputable sources shows the need for health literacy. The results demonstrated that the people receiving the flu vaccine regularly intended to get the COVID-19 vaccination.

In the current study, ages and sex did not have any correlations with the intention to get vaccinated, but a study in France found otherwise (Montagni et al., 2021), which may have been due to the time and place of the study and differences in participant demographics.

The current study also found that the people with low economic status were doubtful about the vaccination, which was consistent with other studies (Edwards et al., 2021; Rhodes et al., 2021). When people are skeptical of information, making decisions about whether to vaccinate could be more difficult.

We also found that people with a history of chronic diseases were doubtful about the vaccination. The reason may be attributed to fear of exacerbation of the disease or death from vaccination. Another important factor from the present study is that people with a skill in correctly using online networks were more eager to get vaccinated. This may be due to their ability to obtain health-related information from the appropriate sources, thereby gaining a greater understanding of the benefits and necessity of the COVID-19 vaccination, leading to an increase in their tendency to get vaccinated (An et al., 2021).

The present study also revealed that the biggest concern of people who did not want to get vaccinated for COVID-19 was the fear of side effects. Other studies were also consistent with this research (Eibensteiner et al., 2021; Marco-Franco et al., 2021). This may be due to the emergence of the COVID-19 disease itself as well as the existence of complex and contradictory information about COVID-19, low e-Health literacy, and the impact of fake news on vaccination complications, which all make some individuals less trusting of the vaccine.

## Study Limitations

The present study was cross-sectional; hence, no causality can be assumed among the variables. Participants in this study were referred to the health centers, which may not be representative of the general population. It is necessary to repeat the survey, particularly with a bigger sample size to confirm the findings for better estimates. The applied tools were self-reported; hence, the intention to get vaccinated against COVID-19 might be overestimated. Future studies should include individuals, such as older adults and those living in rural areas. Since the questionnaire was completed in person and during the COVID-19 outbreak, the low number of people presenting to health centers due to fear of contracting the disease is another limitation. Additionally, the study was conducted only for people who were literate; hence, the research results may be biased based on a population with literacy because the study was conducted via a paper questionnaire. Also, a low knowledge score may indicate that participants misunderstood the question or reflects a bias of social utility.

## Conclusion

According to the results, acceptance of the COVID-19 vaccine is associated with the ability to detect false and misleading information and e-Health literacy. Existence of a high percentage of people who suspect vaccination highlight the need to pay attention to fake news and call for improved e-Health literacy to better guide information on both social media and the news media. The biggest concern of people about not wanting to get the COVID-19 vaccination was the fear of side effects. These findings can be a strong basis for policymakers and health professionals to design and to implement programs to fight the spread of false and misleading information related to the COVID-19 vaccination.
